# Combined effects of *Bacillus* sp. M6 strain and *Sedum alfredii* on rhizosphere community and bioremediation of cadmium polluted soils

**DOI:** 10.3389/fpls.2022.913787

**Published:** 2022-09-21

**Authors:** Abbas Ali Abid, Gengmiao Zhang, Dan He, Huanhe Wang, Itrat Batool, Hongjie Di, Qichun Zhang

**Affiliations:** ^1^Zhejiang Provincial Key Laboratory of Agricultural Resources and Environment, Key Laboratory of Environment Remediation and Ecological Health, Ministry of Education, Zhejiang University, Hangzhou, China; ^2^Zhuji Agricultural Technology Extension Center, Zhuji, China; ^3^Zhuji Economic Speciality Station, Zhuji, China; ^4^Institute of Food Science, Chinese Academy of Agricultural Sciences, Beijing, China

**Keywords:** *Sedum alfredii*, *Bacillus* sp. M6, cadmium, soil microbial community, biochar (BC), bioremediation, rhamnolipid

## Abstract

Concerns regarding inevitable soil translocation and bioaccumulation of cadmium (Cd) in plants have been escalating in concomitance with the posed phytotoxicity and threat to human health. Exhibiting a Cd tolerance, *Bacillus* sp. M6 strain has been reported as a soil amendment owing to its capability of reducing metal bioavailability in soils. The present study investigated the rhizospheric bacterial community of the Cd hyperaccumulator *Sedum alfredii* using 16S *rRNA* gene sequencing. Additionally, the Cd removal efficiency of strain *Bacillus* sp. M6 was enhanced by supplementing with biochar (C), glutamic acid (G), and rhamnolipid (R) to promote the phytoremediation effect of hyperaccumulator *S. alfredii*. To the best of our knowledge, this is the first time the amendments such as C, G, and R together with the plant-microbe system *S. alfredii-Bacillus* sp. M6 has been used for Cd bioremediation. The results showed that soil CaCl_2_ and DTPA (Diethylenetriamine penta-acetic acid) extractable Cd increased by 52.77 and 95.08%, respectively, in all M6 treatments compared to unamended control (CK). *Sedum alfredii* with *Bacillus* sp. M6 supplemented with biochar and rhamnolipid displayed a higher phytoremediation effect, and the removal capability of soil Cd (II) reached up to 16.47%. Moreover, remediation of Cd polluted soil by *Bacillus* sp. M6 also had an impact on the soil microbiome, including ammonia-oxidizing bacteria (AOB), ammonia-oxidizing archaea (AOA), and cadmium transporting ATPase (*cadA)* genes. Quantitative PCR analysis confirmed the *Bacillus* sp. M6 strain increased the abundance of AOB and *cadA* in both low Cd (LC) and high Cd (HC) soils compared to AOA gene abundance. Besides, the abundance of *Proteobacteria* and *Actinobacteria* was found to be highest in both soils representing high tolerance capacity against Cd. While *Firmicutes* ranked third, indicating that the additionof strain could not make it the most dominant species. The results suggested the presence of the hyperaccumulator *S. alfredii* and Cd tolerant strain *Bacillus* sp. M6 supplemented with biochar, and rhamnolipid, play a unique and essential role in the remediation process and reducing the bioavailability of Cd.

## Graphical Abstract

LC, low cadmium; HC, high cadmium; C, biochar; R, rhamnolipid; G: glutamic acid. 
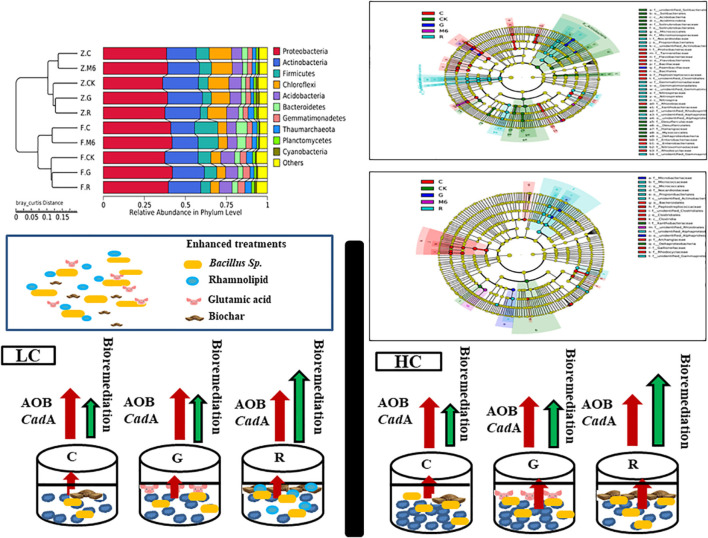

## Introduction

As the intensification of anthropogenic activities such as fertilization, mining, sewage sludge, pesticide abuse, and metallurgy continues to evolve in the social economy it has consequently contributed to soil pollution in the form of heavy metals (Zhou et al., 2016). Cadmium (Cd) contamination of soil is becoming a ubiquitous environmental problem due to uncontrolled industrialization, unsustainable urbanization, and intensive agricultural practices. Cadmium is a toxic element, and severely threatens food safety, human health, and soil quality. As a matter of fact, the soil is exploited as an ultimate source of waste disposal and utilization. The activities in different natural and anthropogenic sources release Cd in the soil, which bio-accumulates in food crops. In China, at least 2 × 10^5^ km^2^ area of farmland (one-fifth of the farmland) has been polluted by Cd (Fan et al., 2013). The mean soil Cd concentrations were found at 1.698 mg kg^–1^ in Zhangshi, an irrigated area of Shenyang (Sun et al., 2006), 0.35 mg kg^–1^ in Shanghai (Hu et al., 2004), 0.8 mg kg^–1^ in Hangzhou (Zhang and Ke, 2004), 0.04 mg kg^–1^ in Guangdong Province (Zhang et al., 2011), and 1.152 mg kg^–1^ in Tianjin (Wu and Cao, 2010). The presence of Cd is quite alarming for the cumulative soil environment because Cd is toxic to plants even at very low concentrations (0.5 μg Cd g^–1^ soil).

Reportedly, Cd influences nitrogen cycling microbial biomass in the soil (Liu et al., 2012a,b). Afzal et al. (2019) reported that nitrifying *amoA* gene abundance in paddy soils was reduced by adding Cd to 5 and 10 mg kg^–1^. Smolders et al. (2001) found that microbially mediated nitrification is suppressed up to 80% by adding Cd in the form of CdCl_2_ (200 mg kg^–1^). Similarly, Khan et al. (2010) confirmed a decrease in microbial enzyme urea amidohydrolase and acid phosphatase activities by up to 33.0% after 2 weeks of soil incubation with 5 mg Cd kg^–1^. Another study by Liu et al. (2018) showed that Cd decreases *amoA* gene abundance in the soils, reducing ammonium oxidation. Like nitrifiers, the denitrifying bacterial community was also sensitive to Cd, significantly impacting the denitrifier community structure (Afzal et al., 2019).

A plethora of heavy metal remediation techniques, such as phytoremediation, stabilization, solidification, electroremediation, and excavation, have been investigated under both controlled and field conditions (Mulligan et al., 2001). Among these techniques, the method of phytoremediation is frequently applied because it exhibits great potential for cost-effective performance and is environment-friendly. However, phytoremediation is mainly affected by the time for phytoremediation and plants’ physiological damage under high-stress conditions. Organic acids can chelate free heavy metal ions, reduce their bioavailability in the soil and eventually reduce their toxic effect (Yao et al., 2022). Besides, some organic acids can also mobilize heavy metals around the roots, making them easier to absorb by plants and achieve a cumulative effect. Biosurfactants, such as rhamnolipids, produced by microorganisms, i.e., *Pseudomonas aeruginosa*, play an essential role in reducing heavy metal toxicity in soil (Juwarkar et al., 2007). Rhamnolipid-mediated bioremediation is one of the frequently proposed potential strategies in the case of indigenous soil bacteria (Mulligan et al., 2001). Glutamic acid is another non-toxic amino acid produced by *Bacillus* species, which has been widely used in heavy metals adsorption (Lei et al., 2016; Luo et al., 2016). It contains several functional groups (-COOH), which can bind metal ions. Alternatively, biochar has a specific structure, and a high surface area can sorb contaminants and heavy metals (Tang et al., 2013).

*Sedum alfredii* has attracted increasing attention from researchers in China due to its distinct Cd co-hyperaccumulator tendencies (Yang et al., 2004a,b). The prevailing paradigm showed that metal-tolerant bacteria isolated from *S. alfredii* rhizosphere significantly enhanced Cd extraction (Pan et al., 2017). However, the basic ecology of indigenous soil microbial communities associated with the phytoextraction process in polluted soils by plant species needs further appraisal. In this study, a strain of Cd tolerant *Bacillus* sp. M6, isolated previously by Zhou et al. (2017), was used to mobilize heavy metals (mobilization capacity > 30%) by different carrier loading to increase the bioavailability of Cd in *S. alfredii* soil. For the first time, *Bacillus* sp. M6 was supplemented with biochar, glutamic acid, and rhamnolipid amendments to enhance the activity for Cd bioremediation. The effects of all these biochar, glutamic acid, and rhamnolipid amendments combined with *Bacillus* sp. M6 strain apropos of Cd remediation necessitates further exploration. Hence, the objectives of the present study are: (1) to explore the feasibility of Cd tolerant strain *Bacillus* sp. M6 as an inoculum to detoxify Cd; (2) to observe if soil inoculation with *Bacillus* sp. M6 and some amendments increase the efficiency of Cd removal from soil; (3) to analyze the composition and functionality of bacterial rhizobium upon inoculation; and (4) to evaluate the accumulation potential of Cd in *S. alfredii*.

## Materials and methods

### Soil sampling

To explore the phytoremediation strategies of Cd contaminated soil, low Cd contaminated (LC) and high Cd contaminated (HC) agricultural soils were sampled. The soils with Cd contents < 0.70 mg kg^–1^ (considered low-contaminated soils) were collected from Zhuji County of Shaoxing, Zhejiang Province, China. The study area belongs to a subtropical humid monsoon climate with an average annual temperature of 16.3?, having four distinct seasons, more rain, and sufficient light. The mean annual precipitation is about 1,373.6 mm, and the mean annual sunshine is about 1,887.6 h. The soils with Cd contents of 0.70 mg kg^–1^ or more were considered highly polluted soils, and HC was collected from farmland in the Fuyang District of Hangzhou City, Zhejiang Province. The sampling site of the Fuyang District has a subtropical monsoon climate, with an average annual temperature of 16.10?, annual average sunshine of 1,927.7 h, and a frost-free period of 231 days, and an annual rainfall of 1,441.9 mm. Random soil samples were collected from 0 to 20 cm of soil surface. The soil cores were packed in sterile plastic bags, sealed, and transported to the laboratory. Each sample was divided, and one subsample was incubated for the remediation experiment, while another sample was sieved through a 2.0 mm mesh for subsequent physicochemical analysis (Table 1).

**TABLE 1:** Basic physical and chemical properties of soil.

Soils	AP mg kg^–1^	AK mg kg^–1^	AN mg kg^–1^	OM mg kg^–1^	pH	Total Cd mg kg^–1^
HC	13.38	146.67	162.96	63.67	8.13	4.74
LC	13.92	135.70	264.00	76.00	7.07	0.70

HC, high cadmium (Cd) contaminated soil (>0.70 mg/kg); LC, low Cd contaminated soils (<0.70 mg/kg); AP, available phosphorus; AK, available potassium; AN, alkali hydrolyzed nitrogen; OM, organic matter.

### Microbial inoculum preparation

The isolated strain *Bacillus* sp. M6 with minimum inhibitory concentration (MIC) 100 mg L^–1^ CdCl_2_ was cultured in lysogeny broth (LB medium) at 28°C. The preliminary experiment indicated the strain has the increasing capability of Cd (II) biosorption and bioaccumulation (Zhou et al., 2017). The strain was then deposited in the NCBI gene bank^1^ with accession number KM349307. The cells were harvested after culturing in LB medium for 48 h at 28°C, at the mid-log phase of the growth curve, showing the optical density (microbial concentration/absorbance) of 0.1–1.2 at 600 nm to ensure cfu ≥ 1 × 10^8^ cells/ml. After harvesting, the cells were centrifuged (5,000 rpm) at 4°C for 10 min, washed five times with deionized water, and re-suspended in a 0.9% NaCl solution. The strain was supplemented by (1) 1 g sterilized pine bamboo biochar (prepared in an oxygen-free closed container at 600? for 2 h), and 35 ml re-suspended strain were mixed and shaken at 160 rpm at 25°C for 6 h; (2) 1 g glutamic acid and 35 ml re-suspended strain were mixed and shaken at 160 rpm at 25°C for 6 h; (3) 1 mmol rhamnolipid (Ruijie Biology) and 35 ml re-suspended strain were mixed and shaken at 160 rpm at 25°C for 6 h.

### Morphology by scanning electron microscopy

The effects of amendments on the structural morphology of the M6 strain were studied using the scanning electron microscopic (SEM) method. The supernatant was removed from the bacterial suspension by centrifugation. A small amount of the sample was wrapped with filter paper and placed overnight in a 2.5% glutaraldehyde solution at 4°C. After that; the samples were rinsed three times for 15 min with 0.1 M phosphoric acid buffer solution having pH 7. The samples were treated with 1% osmic acid solution for 2 h and rinsed again with phosphoric acid buffer solution. The samples were dehydrated with gradient concentration ethanol (including 30, 50, 70, 80, 90, and 95%) for 15 min and then dehydrated by 100% ethanol for 20 min and repeated two times. Finally, the samples were treated with a mixture of ethanol and isoamyl acetate (v/v = 1:1) for 30 min and then treated with pure isoamyl acetate. After keeping it overnight, the samples were dried at a critical limit. Finally, the treated samples were observed using a Hitachi TM-1,000 scanning electron microscope.

### Pot experimental design/layout

A pot experiment was conducted to evaluate the effect of Cd on the soil microbial community. A PVC plastic pot with a diameter of 20 cm × 15 cm was used in the experiment. About 2.5 kg of air-dried soil was weighed and placed into the pots. The soil water contents were adjusted to 60% field capacity (FC), and five *S. alfredii* plants were transferred to each pot. Afterward, different bacterial suspensions were incorporated. Five sets of each treatment, including (1) *Bacillus* sp. M6 (M6); (2) *Bacillus* sp. M6 plus biochar (C); (3) *Bacillus* sp. M6 plus glutamic acid (G); (4) *Bacillus* sp. M6 plus rhamnolipid (R) were prepared, in addition, to control having ddH_2_O (CK). Each treatment had three further replicates. The different bacterial suspensions were poured into the roots of *S. alfredii* plant growing in the corresponding pot at a rate of 150 ml per pot after every 10 days. The *S. alfredii* plants were harvested after applying five doses of the bacterial suspension. After harvesting the plant, fresh soil samples were collected from each pot. Each replicated soil sample was then divided, and one subsample was stored at –80°C for subsequent microbial analysis. The other soil sample was sieved through a 2.0 mm mesh for subsequent analysis of chemical properties.

### Plant sample analysis and soil characterization

Soil pH was measured with a pH meter (1:25) and soil available Cd contents were determined using graphite furnace atomic absorption spectrophotometer (PE AA800) with CaCl_2_ extraction. Soil ammonium and nitrate content were determined using the KCL extraction method using a continuous flow analyzer (SAN + + Skalar. Holland). The stems and leaves of *S. alfredii* were separated, washed with tap water followed by deionized water, and placed in the oven for 30 min at a temperature of 105°C to deactivate the enzymes. After obtaining the constant weight at 70°C, the plants were ground with a ball mill (JXFSTPRP-II, Shanghai Jingxin Industrial Development Co., Ltd.), passed through a 100 mm sieve then stored in the plastic zipper bag. Total Cd content in plants was determined by graphite furnace atomic absorption spectrophotometer (PE AA800) with microwave digestion method.

### Extraction of soil DNA and quantitative polymerase chain reaction of functional genes

The Fast DNA Spin Kit was used to extract soil DNA from 0.5 g fresh soil samples according to the manufacturer’s instructions (MP Biomedicals, LLC, Solon, OH, United States). The size and integrity of DNA were examined by 0.7% agarose gel electrophoresis, and the quality and purity of DNA were analyzed by NanoDrop ND-2000 UV-vis (NanoDrop Technologies, Wilmington, DE, United States). The extracted DNA was stored at –20°C for further microbial analyses.

The ammonia-oxidizing bacteria (AOB), ammonia-oxidizing archaea (AOA), and cadmium transporting ATPase (*cadA)* genes were quantified by quantitative PCR (qPCR) according to Muema et al. (2015). The qPCR was performed using Light Cycler 480 (Roche, Germany). A total of 20 μl reaction mixture was used for the amplification, including 10 μl of SYBR Premix Ex Taq (Takara, Dalian, China), 1 μl of DNA template (1–10 ng), 1 μl of each primer (10 μM), and added sterilized ddH_2_O 7 μl (Supplementary Table 1). The primers of each target gene and thermal cycling conditions are given in Supplementary Table 1. The standards of the targeted genes AOB, AOA, and *cadA* genes were prepared using the positive clones isolated from the plasmid. Plasmid DNA concentration was determined by NanoDrop 2,000 UV-Vis Spectrophotometer (NanoDrop Technologies, Wilmington, DE, United States) by calculating the AOB, AOA, and *cadA* genes copy numbers. Ten-fold serial dilutions were performed to generate the standard curve of the plasmid DNA. Clones with efficiency and correlation coefficients above 95% and 0.98, respectively, were employed to make the standard curve.

### Bacterial 16S amplification and high throughput sequencing

The extracted DNA of soil samples was sequenced using the V4 region of 16S rRNA by Novogene, Shanghai, China.^2^ The primers 515F (5’-GTGCCAGCMGCCGCGGTAA-3’) and 806R (5’-G GACTACHVGGGTWTCTAAT-3’) were used for bacterial community analysis. High-Fidelity polymerase chain reaction (PCR) Master Mix with GC Buffer was purchased from New England Biolabs’ Phusion. High-efficiency and high-fidelity enzymes were used for PCR to ensure the efficiency and accuracy of amplification. The PCR mixture contained a 30 μl reaction volume, including 15 μl Phusion Master Mix (2 ×), 3 μl primer (2 μM), 15 μl gDNA (1 ng/μl), and 0 μl of sterilized ddH_2_O. The amplification was done for 1 min at 98°C, followed by 30 cycles of 10 s at 98°C, 30 s at 50°C, and 30 s at 72°C. The PCR products were then purified by 2% agarose gel electrophoresis. The band size of 400–450 bp as the target bands was recovered and purified using the GeneJET gel recovery kit (Thermo Scientific). A gene library was constructed using the New England Biolab’s NEB Next Ultra DNA Library Prep Kit. After Qubit quantification and library detection, the library was sequenced by HiSeq 2,500 PE250. QIIME 1.9.1 was used to filter, optimize, and control the sequence information. Finally, 97% of sequences with similar levels were classified into one OTUs. In addition, cluster analysis was performed based on calculated Bray-Curtis dissimilarities between the samples. The specific bacterial taxa were recognized separately by LEfSe from phylum to genus level.

### Statistical data analysis

The standard errors and means of the data were computed using Excel (Microsoft, United States). The significance of the difference between the treatments was performed by one-way analysis of variance (ANOVA) using SPSS 20.0 (SPSS Inc., United States). The results were considered significant at *p* < 0.05. Graphs were prepared by using Origin Pro version 2021 (Origin lab corporation, Wellesley Hills, Wellesley, MA, United States).

## Results and discussion

The hyperaccumulator *S. alfredii* not only tolerates high levels of Cd but also efficiently takes up and accumulates large amounts of Cd in its aboveground parts (Ge et al., 2021), and thereby could prove to be useful in the phytoremediation of Cd polluted soils (Yang et al., 2004a). Besides, rhamnolipids are bio-surfactants produced by microorganisms, i.e., *Pseudomonas aeruginosa*, with the potential to mobilize heavy metals in the soil (Mulligan et al., 2001; Juwarkar et al., 2007). A Cd tolerant *Bacillus* sp. M6 strain was used, with a tolerance capacity of 14.1% toward Cd (Zhou et al., 2017). The inoculation of *Bacillus* can improve the mobility and bioavailability of Cd in soil and increases plants’ extraction rate by two times. In this study, we found that the addition of *Bacillus* sp. M6 could effectively increase the exchangeable state of Cd in the rhizosphere soil of *S. alfredii* (Jeong et al., 2012; Mo et al., 2022). Since this remains challenging how the supplemented Cd tolerant *Bacillus* sp. M6 strain influences the rhizosphere community composition and activity in Cd contaminated soils. Therefore, a 16 S high-throughput sequencing approach was applied to gain insights into the rhizosphere microbial community composition that may influence metal accumulation in the hyperaccumulator *S. alfredii*.

### Morphology of the supplemented strain

**FIGURE 1:**
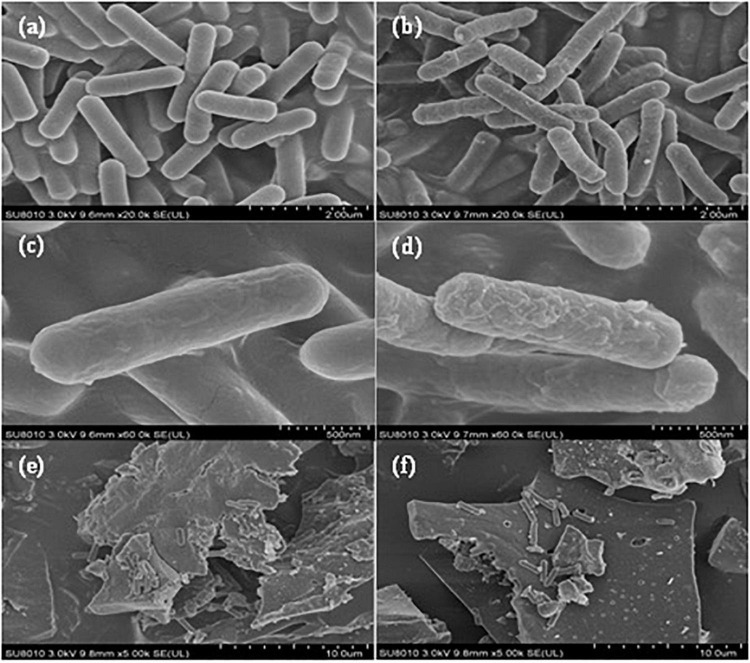
The bacterial structure of electron microscopy by using *Bacillus* sp. M6. And amendments **(a)** M6 strain 2 μm scale; **(b)** M6 + rhamnolipid 2 μm scale; **(c)** M6 strain 500 nm scale; **(d)** M6 + rhamnolipid 500 nm scale; **(e,f)** M6 + biochar.

The surface membrane of bacteria under *Bacillus* sp. M6 strain supplemented with rhamnolipid was rough (Figures 1b,d) in contrast to the bacteria without rhamnolipid (Figures 1a,c), the surface membrane was smooth, indicating rhamnolipid impact on the permeability of the bacterial surface membrane. The same results were found by Ragheb et al. (2000) demonstrated rhamnolipid as a biosurfactant, mobilizes heavy metals in soil by metal desorption from rhizosphere through complexation, ion exchange, or accumulation on the surface by reducing interfacial tension (Lal et al., 2018; Mishra et al., 2021) and changes the membrane permeability of bacteria that could help bacteria to release organic acids and enhance the activation effect. The strain *Bacillus* sp. M6 adsorbed significantly in the gaps and on the surface of biochar, respectively (Figures 1e,f). A porous microstructure and lacunose surfaces of biochar provide habitats for bacteria. Moreover, the soluble organic carbon and other nutrients adsorbed by biochar can provide substrates for the survival of microorganisms, thus improving the abundance and activity of microbes in the soil (Atkinson et al., 2010; Beesley and Marmiroli, 2011).

### Effects of the supplemented strain on soil pH and available Cd content

In order to precisely predict metal availability in soils, we use CaCl_2_ and DTPA (diethylenetriaminepentaacetic acid) extractions measurements to assess metal availability in soils (Hseu and Lai, 2017). The availability of the metals in soils cannot be precisely predicted unless we measure CaCl_2_ and DTPA extractions (Hseu and Lai, 2017). Moreover, the metals adsorbed on organic matter are potentially available and can be extracted by DTPA. Hence, it is important to extract DTPA-Cd to make our results more precise.

**FIGURE 2:**
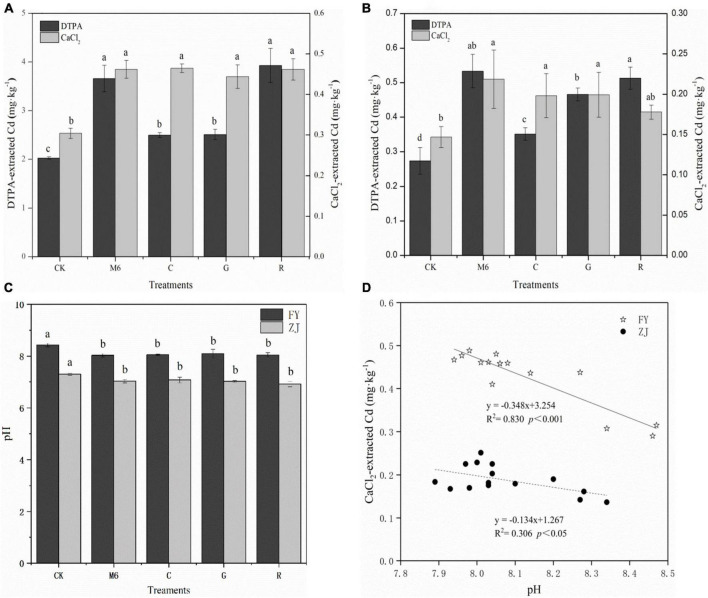
Changes in pH and available cadmium in LC and HC soils. **(A)** Changes in extractable Cd of DTPA and CaCl_2_ in HC soils; **(B)** changes in extractable Cd of DTPA and CaCl_2_ in LC soils; **(C)** changes in soil pH; **(D)** correlation between soil pH and CaCl_2_-extracted Cd. The arrow bar above the line shows standard error. The different letters show significant differences while same letters represent no significant differences among the treatments. CK, control; M6, M6 strain; C, M6 + Biochar; G, M6 + Glutamic acid; R, M6 + rhamnolipid; FY, Fuyang; ZJ, Zhuji; LC, low cadmium; HC, high cadmium; DTPA, Diethyene triaminepenta acetic acid.

The CaCl_2_ and DTPA extractable Cd were significantly affected by the supplemented strain. In HC soils, CaCl_2_ extractable Cd increased by 45.91∼52.77% in all supplemented treatments compared to CK. However, no significant difference among the supplemented treatments was found (Figure 2A). Besides, DTPA extractable Cd content was enhanced by 23.31∼93.84% compared to CK, and the increase in treatments M6 and R was recorded as significantly higher than that of C and G treatments in both high and low Cd soils (Figures 2A,B). In LC soils, CaCl_2_ extractable Cd increased significantly by 21.14∼48.98% in all supplemented treatments compared to CK. While comparing supplemented treatments, M6 promoted the most CaCl_2_ extractable Cd (Figure 2B). Besides, DTPA extractable Cd content in LC soils increased by 28.52∼95.08%, and the highest increase was observed in treatments M6 and R. The same results were found in HC soils, indicating that rhamnolipid promoted extractable Cd because of its biosurfactant, which has less toxicity than an active chemical agent and can degrade itself in soil due to its activation effect (Zhao et al., 2018). In addition, it can enhance the migration ability of heavy metals by reducing the interfacial tension between heavy metals and soil (Wang and Mulligan, 2004).

It has been found that *Bacillus* sp. M6 strain can reduce soil pH to enhance soil Cd availability (Zhou et al., 2017). Compared to CK, the pH of all treatments decreased significantly in both soils (Figure 2C). The soil pH influences the solubility of heavy metals and the ionization state of numerous functional groups on a large surface area (Argun et al., 2009). At low pH, large amounts of H + ion cause strong electrostatic repulsion, which decreases the binding of Cd (II) by carboxyl groups and other acidic functional groups. A significant negative correlation between CaCl_2_ extractable Cd and pH in LC and HC soils, respectively, was found (*p* < 0.05, *p* < 0.001) (Figure 2D), which is in accordance with the previous studies (McBride, 2002; Römkens et al., 2011). After amendment application, the pH of the soil was reduced, and the amount of extractable Cd was improved, favoring the accumulation of Cd by hyper-accumulators.

### Effect of the different supplemented strains on soil functional gene abundance

**FIGURE 3:**
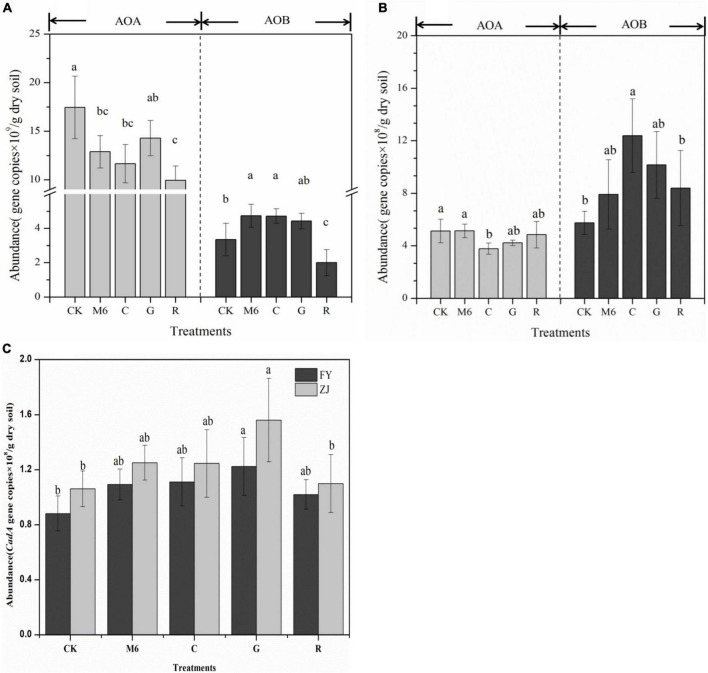
Abundance of soil functional genes *amoA* AOA and *amoA* AOB in **(A)** HC **(B)** LC soils collected from two different soils. **(C)** The abundance of *cadA* genes in two soils. Data points represent means the standard deviations (*n* = 3). The various lowercase letters indicate significant differences among all treatments at *p* < 0.05. CK, control; M6, M6 strain; C, M6 + Biochar; G, M6 + Glutamic acid; R, M6 + rhamnolipid; FY, Fuyang County; ZJ, Zhuji County; LC, low cadmium; HC, high cadmium.

The introduction of *Bacillus* sp. M6 strain increased the abundance of AOB in all supplemented treatments, while the AOA gene abundance was decreased in all supplemented treatments compared to CK (Figures 3A,B). In HC soils, the AOA gene abundance ranged from 0.99 × 10^10^-1.74 × 10^10^ copies/g in dry soils. The AOA gene abundance in M6, C, and R treatments was significantly decreased, and the lowest abundance was observed in R treatment (Figure 3A). Although, rhamnolipid can mobilize soil heavy metals by itself. It also changes the membrane permeability of some bacteria, which may negatively impact the growth and survival of some bacteria. In LC soils, the AOA gene abundance was 3.78 × 10^8^ – 5.13 × 10^8^ copies/g in dry soil, and the decrease was not evident compared to CK, except in the biochar treatment (Figure 3B). Interestingly, the AOB gene abundance increased significantly in all supplemented treatments than CK, indicating the abundance of nitrifying bacteria being increased with the addition of exogenous bacteria. This AOB increase might be due to the introduction of exogenous microbial agents, increasing soil mineralization. The active carbon and nitrogen improved by mineralization can promote *amoA* AOB growth (Liu et al., 2018), whereas *amoA* AOA plays a role in the soil environment with low nitrogen content. Also, more bacterial activity was found in low contaminated soil than with high contamination because of the growth restrictions by high Cd concentration manifesting as the inhibition of cell growth and cell detoxification mechanism (i.e., intracellular efflux of Cd^+2^).

The *cadA* functional gene can encode P-type ATPase in the *cad-ABC* system of Gram-positive bacteria. The strain can pump Cd out of the bacteria through the enzyme encoded by the *cadA* gene to achieve detoxification (Tsai et al., 1992; Sebastian et al., 2019). The abundance of *cadA* gene in HC and LC soils ranged from 8.81 × 10^7^-1.22 × 10^8^ copies/g dry soil and 1.06 × 10^8^-1.56 × 10^8^ copies/g dry soil, respectively (Figure 3C). It was found that the *cadA* gene abundance in all supplemented treatments was increased compared to CK, which was beneficial in enhancing the resistance of soil microorganisms to Cd. The *cadA* abundance of G treatment was significantly increased by 38.48 and 47.17% in Fuyang and Zhuji, respectively, than that of the CK treatment, indicating that glutamic acid was more conducive to bacterial growth.

### Effect of the supplemented strain on soil microbial community

The soil microbial community is the leading player in heavy metal remediation in the soil. Also, plant growth and the availability of heavy metals can be stimulated by microbial communities. Hence, understanding the basic ecology of soil communities associated with plant remediation is very important to optimize the plant remediation process (Wood et al., 2016). The results showed the soil microbial community was affected to a certain extent after applying different treatments. The abundance of *Proteobacteria* and *Actinobacteria* was found highest in both soils (Supplementary Figures 1a,b), showing high tolerance capacity against Cd (Liu et al., 2020a,b). While *Firmicutes* ranked third, indicating the addition of strain could not make it the most dominant species. Most of the heavy metal-resistant genes are associated with phyla *Firmicutes*, and that is why phyla *Firmicutes* can promote their adaptation to heavy metal polluted environments (Guo et al., 2019). Contradictory to that, the phyla *Firmicutes* growth in our study was not higher than *Proteobacteria* and *Actinobacteria*. The reason might be due to increasing bacterial suspension doses (five doses). Hence, the strain was no more competitive in the rhizosphere, since the *Firmicutes* population is not the most relevant taxa upon repeated inoculation. In HC soils, the relative abundance of *Firmicutes* ranged from 5.91 to 14.16% (Supplementary Figure 1A). Moreover, the abundance of *Firmicutes* in R treatment was reduced relatively. In contrast, the highest abundance was found in the C treatment, indicating that biochar had promoted the survival efficiency of *Firmicutes* while glycolipids reduced the survival efficiency of *Firmicutes*. A similar trend of *Firmicutes* abundance was found In LC soils (Supplementary Figure 1B). The number of OTUs from low to high was represented by blue to red (Supplementary Figure 1A), and it can be observed clearly that the abundance of *Firmicutes* in the C and M6 treatment was high. The rationale for the high abundance of *Firmicutes* in C treatment was due to adding biochar as a sole carbon source. The phospholipid bilayer structure on the cell surface of the strain was more compatible with rhamnolipid. After long-term Cd addition, the rhamnolipid may damage the membrane structure and affect its uptake and assimilation mechanism (Kuyukina et al., 2005). Subsequently, the porous structure of biochar facilitates the growth of bacteria and changes microbial community structure in soil (Wang et al., 2018). A previous study has also shown that biochar can affect soil microbial community composition by regulating the redox process of heavy metal ions (Chen et al., 2016).

**FIGURE 4:**
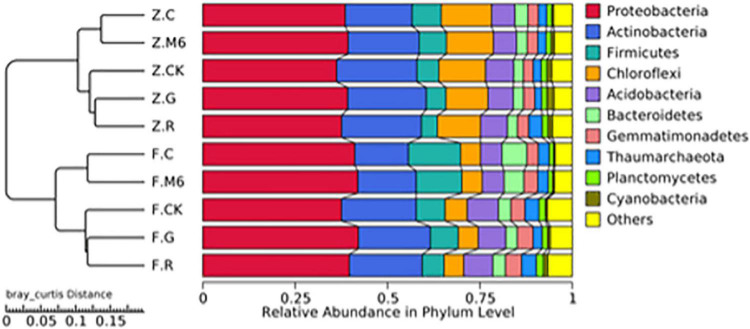
The cluster dendrogram of Bray-Curtis dissimilarity of samples. CK, control; M6, M6 strain; C, M6 + Biochar; G, M6 + Glutamic acid; R, M6 + rhamnolipid; F, Fuyang County; Z, Zhuji County.

**FIGURE 5:**
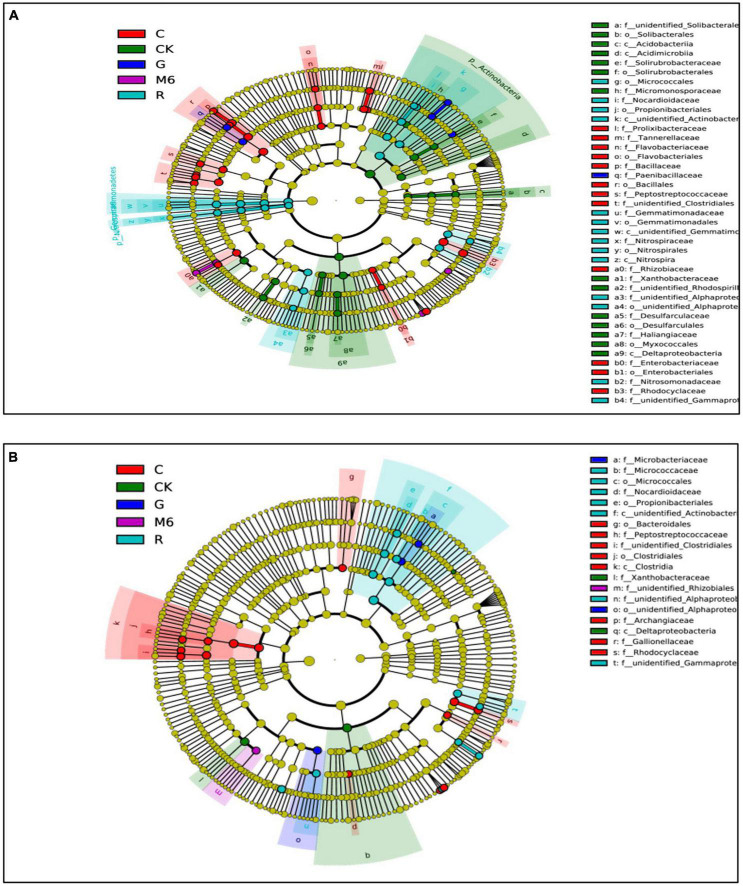
LEfSe cladogram indicating the phylogenetic distribution of bacteria lineages under different treatments in HC **(A)** and LC **(B)** soils. In the figure, red, green, dark blue, purple, and light blue circles, respectively represent the flora of C, CK, G, M6, and R treatments which are significantly higher than those of the other four treatments. LDA > 3 is taken to represent the significant difference, and the circles from inside to outside, respectively represent the classification level of domain, phylum, class, order, family, genus, and species. CK, control; M6, M6 strain; C, M6 + Biochar; G, M6 + Glutamic acid; R, M6 + rhamnolipid; FY, Fuyang County; ZJ, Zhuji County; LC, low cadmium; HC, high cadmium; LEfSe, Linear discriminant analysis effect size; LDA, Linear discriminant analysis.

The cluster analysis of relative abundance by Bray Curtis distance showed the bacterial community relative abundance was different in C and M6 treatments from that of the un-amended control (Figure 4). Linear discriminant analysis (LEfSe) was further used to study the effects of different treatments on soil microbial abundance at different classification levels (Figure 5). The LDA > 3.0 was chosen as a significant difference in abundance. In HC soil, the abundance of 24 differentiated branches increased in C treatment from the phylum level to the species level, which was the highest among all treatments. The same results were found in LC soil with a total of 14 branches. Besides, the biochar with strain *Bacillus* sp. M6 exhibited a significant response in HC soil from order to genus level, suggesting that biochar treatment positively affected the relative activity of the target strain, more evidently in HC soils. The biochar has an advantage over other amendments because it has large porosity, specific surface area, and abundant oxygen-containing functional groups on the surface. Also, because of the strong electrostatic field and adsorption reaction, it can bind small and charged particles, having a size < 0.25 mm, to form larger aggregates (Wang et al., 2019). Besides, the effect of biochar on soil microbial and enzyme activities might enhance the soil organic matter storage significantly and improve soil aggregate stability (Tang et al., 2022).

### Cadmium contents of *Sedum alfredii* under different treatments

**FIGURE 6:**
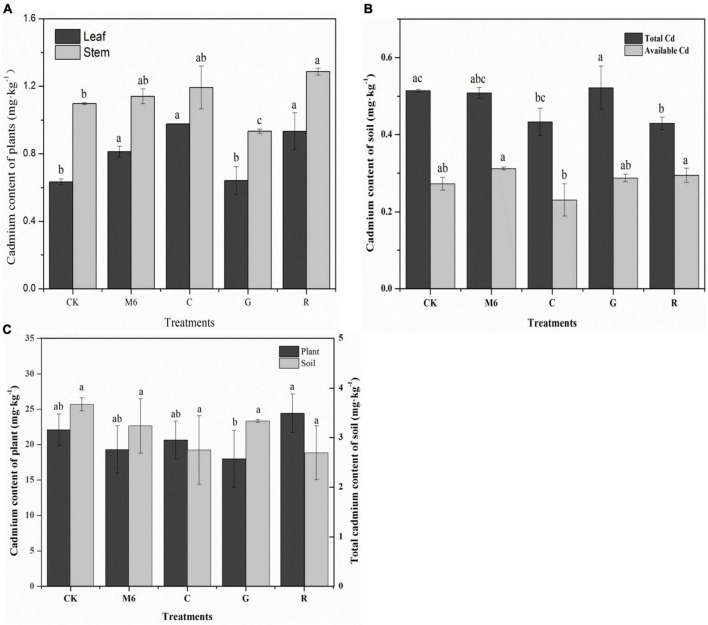
Cd content in stems and leaves of *Sedum alfredii* **(A)** and total Cd and available Cd content in LC soil **(B)**, Cd content of *Sedum alfredii* and total soil Cd content in HC **(C)**. The data is the means and standard deviations of three replicates (*n* = 3). The significant differences among all treatments (*p* < 0.05) are presented by lowercase letters. LC, low cadmium; HC, high cadmium.

It was found that *S. alfredii* has the ability to absorb more Cd, which in turn led to a decrease of Cd concentration in the rhizosphere, in line with the results of Tang et al. (2020), who revealed in a field trial that *S. alfredii* uptake Cd from the rhizosphere. Hou et al. (2018) confirmed after 150 days of the experiment that *S. alfredii* exhibited a significant difference in metal tolerance and accumulation in Cd contaminated soils (Hou et al., 2018). After the addition of treatments, Cd level increased in the *S. alfredii* leaves compared to CK, especially under C and R treatments, increased by 54.05 and 47.11%, respectively (Figure 6A), showing that C and R treatments promoted phytoremediation. However, Cd content in leaf and stem was significantly reduced in G treatment compared to CK. The inoculation of Cd-resistant bacteria, which is immobilized by biochar, significantly increased the accumulation of Cd in the stem and roots of the plant (Chuaphasuk and Prapagdee, 2019). Li et al. (2007) found that the combined effects of bacteria and *S. alfredii* increased the accumulation of heavy metals in plants, which was inconsistent with our results. In stem, significantly higher Cd accumulation was found in R and C treatments compared to CK. The total plant Cd content in R was 3.58 mg kg^–1^, which was higher than CK, while total soil Cd content in C and R treatments was found to be lowest (Figure 6C). Among C and R, comparatively lower Cd contents in the soils was found in R treatment (0.43 mg kg^–1^), significantly lower than that of CK by 16.47% (Figure 6C).

Under the influence of *S. alfredii* root absorption, the soil available Cd changed significantly (Figure 6B). Soil available Cd in C treatments decreased significantly compared to CK, while a 14.33% increase was observed in M6 treatment compared to CK (Figure 6B). However, a significant increase was observed in Cd content of *S. alfredii* plants in R treatment, and the increase was 10.59% in comparison with CK in HC soils (Figure 6C). The results also reveal that the total soil Cd content decreased in all treatments, especially in C and R treatment, which reduced by 24.60∼26.70% compared with CK. The main reason might be the growth inhibition of strain to some extent, with the increase of Cd pollution, resulting in a reduced activation effect.

## Conclusion

The specific function of strain *Bacillus* sp. M6 was supplemented by bamboo biochar, glutamic and rhamnolipid. A hyperaccumulating plant and a bacterial strain were combined to remediate low and high Cd contaminated soils. The results indicated that soil pH decreased after being treated with supplemented strain and *S. alfredii*. In C treatment, *S. alfredii* showed a good phytoremediation effect, and the total Cd content of the soil was significantly reduced, especially in low Cd contaminated soil. Besides, the microbial abundance of soil nitrogen cycling function was also affected by adding microbial agents. Compared to the control, the Cd accumulation in stems and leaves of *S. alfredii* under R and C treatment was increased. It has been concluded that R and C amendments combined with M6 strain are the best options. Further studies are needed to evaluate the mechanism of Cd accumulation and remediation using the M6 strain with different R and C levels.

## Data availability statement

The data can be extracted from the NCBI gene bank with accession number: KM349307.

## Author contributions

AA: conceptualization, methodology, software, data curation, and writing–original draft preparation. GZ and HD: writing—review and editing. DH: methodology. IB and HW: methodology, writing, and software. QZ: conceptualization, writing—review and editing, validation, funding acquisition, and resources. All authors contributed to the article and approved the submitted version.

## Footnotes

1
https://www.ncbi.nlm.nih.gov

2
www.novogene.com
