# Supplementary material for: Combined effects of Bacillus sp. M6 strain and Sedum alfredii on rhizosphere community and bioremediation of cadmium polluted soils
Source: Front Plant Sci. 2022 Sep 21;13:913787. doi: 10.3389/fpls.2022.913787 (PMC9533712; doi:10.3389/fpls.2022.913787)
Supplement: Supplementary file 1 [file Data_Sheet_1.docx]

**Supplementary material**

**Combined effects of *Bacillus sp.* M6 strain and *Sedum alfredii* on rhizosphere community and bioremediation of cadmium polluted soils**

Abbas Ali Abid^1^, Gengmiao Zhang^2^, Dan He^3^, Huanhe Wang^1^, Itrat Batool^4^, Hongjie Di^1^, Qichun Zhang*^1^

^1^Zhejiang Provincial Key Laboratory of Agricultural Resources and Environment, Key Laboratory of Environment Remediation and Ecological Health, Ministry of Education, Zhejiang University, Hangzhou, 310058, P.R China

^2^Zhuji Agricultural Technology Extension Center, Zhejiang 311800, China

^3^Zhuji economic specialty Station, Zhejiang 311800, China

^4^Institute of Food Science, Chinese Academy of Agricultural Sciences, Beijing

*Corresponding author

Dr. Qichun Zhang

College of Environmental and Resource Sciences

Zhejiang University, Hangzhou, 310058, P.R China

Phone: +86-571-88982413

Email: [qczhang@zju.edu.cn](mailto:qczhang@zju.edu.cn)

**Table S1:** Primers and conditions for real-time PCR analysis

| Functional gene | primers | Primer sequence（5'-3'） | Amplification conditions | reference |
| --- | --- | --- | --- | --- |
| Archaeal *amoA* | Arch-*amoAf* | STAATGGTCTGGCTTAGACG | 2 min at 95℃；40 cycles of 20s at 95℃， 20s at 55℃，30s at 72℃ | Francis et al. 2005 |
|  | Arch-*amoAr* | GCGGCCATCCATCTGTATGT |  |  |
| Bacteria *amoA* | *amoA*1F | GGGGTTTCTACTGGTGGT | 2 min at 95℃；40 cycles of 20s at 95℃，30s at 57℃，30s at 72℃ | Rotthauwe et al. 1997 |
|  | *amoA*2R | CCCCTCKGSAAAGCCTTCTTC |  |  |
| *cadA* | *cad*1F | CAAAYTGYGCRGGHAARTTYGA | 2 min at 95℃；40 cycles of 20s at 95℃，30s at 52℃，30s at 72℃ | Cécile et al. 2001 |
|  | *cad*2R | AACTAATGCACAAGGACA |  |  |


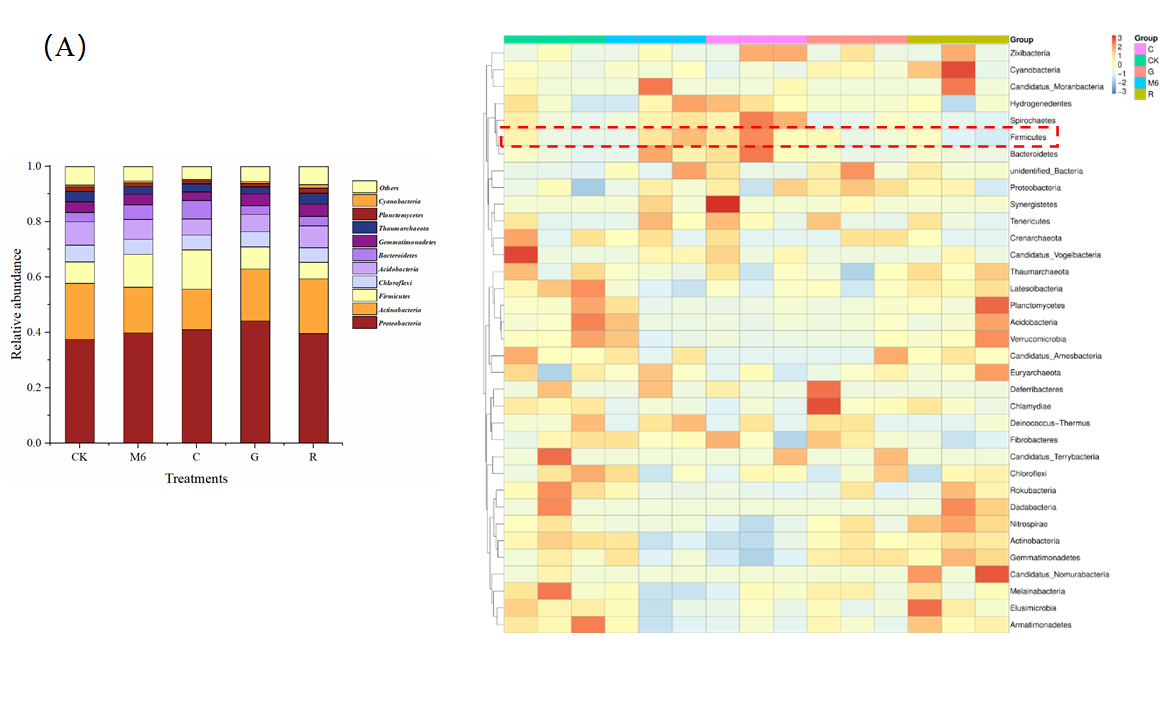


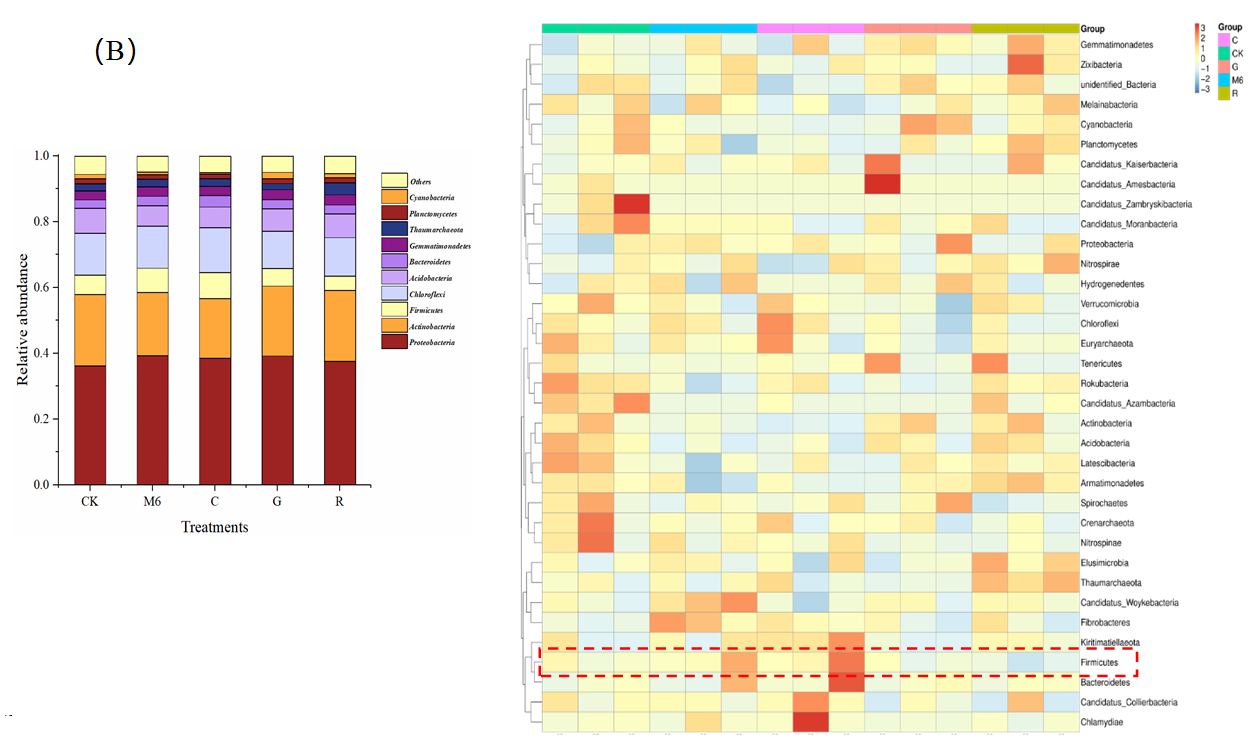


Fig. S1: Proportion of bacterial species in different treatments 670 and heatmap in (A) HC soil and (B) LC soil. The number of OTUs from low to high represented by blue to red

**References**

Francis, C.A., Santoro, A.E., Oakley, B.B. 2005. Ubiquity and diversity of ammonia-oxidizing archaea in water columns and sediments of the ocean [J]. Proceedings of the National Academy of Sciences of the United States of America. 102 (41), 14683-14688.

Rotthauwe, J.H. 1997. The ammonia monooxygenase structural gene *amoA* as a functional marker: molecular fine-scale analysis of natural ammonia-oxidizing populations. Appl. Environ. Microbiol. 63(12), 4704–4712.

Cécile, O., Berthe, T., Quillet, L. 2001. Estimation of the abundance of the cadmium resistance gene *cadA* in microbial communities in polluted estuary water. Res. Microbiol. 152(7), 671-678.
